# Global transcript profiling of transgenic plants constitutively overexpressing the RNA-binding protein *At*GRP7

**DOI:** 10.1186/1471-2229-10-221

**Published:** 2010-10-14

**Authors:** Corinna Streitner, Lars Hennig, Christin Korneli, Dorothee Staiger

**Affiliations:** 1 Molecular Cell Physiology, Bielefeld University, Bielefeld, Germany; 2 Department of Biology & Zurich-Basel Plant Science Center, ETH Zurich, Switzerland; 3 Department of Plant Biology and Forest Genetics, Uppsala BioCenter, Swedish University of Agricultural Sciences, Uppsala, Sweden

## Abstract

**Background:**

The clock-controlled RNA-binding protein *At*GRP7 influences circadian oscillations of its own transcript at the post-transcriptional level. To identify additional targets that are regulated by *At*GRP7, transcript profiles of transgenic plants constitutively overexpressing *At*GRP7 (*At*GRP7-ox) and wild type plants were compared.

**Results:**

Approximately 1.4% of the transcripts represented on the Affymetrix ATH1 microarray showed changes in steady-state abundance upon *At*GRP7 overexpression. One third of the differentially expressed genes are controlled by the circadian clock, and they show a distinct bias of their phase: The up-regulated genes preferentially peak around dawn, roughly opposite to the *At*GRP7 peak abundance whereas the down-regulated genes preferentially peak at the end of the day. Further, transcripts responsive to abiotic and biotic stimuli were enriched among *At*GRP7 targets. Transcripts encoding the pathogenesis-related PR1 and PR2 proteins were elevated in *At*GRP7-ox plants but not in plants overexpressing *At*GRP7 with a point mutation in the RNA-binding domain, indicating that the regulation involves RNA binding activity of *At*GRP7. Gene set enrichment analysis uncovered components involved in ribosome function and RNA metabolism among groups of genes upregulated in *At*GRP7-ox plants, consistent with its role in post-transcriptional regulation.

**Conclusion:**

Apart from regulating a suite of circadian transcripts in a time-of-day dependent manner *At*GRP7, both directly and indirectly, affects other transcripts including transcripts responsive to abiotic and biotic stimuli. This suggests a regulatory role of *At*GRP7 in the output of the endogenous clock and a complex network of transcripts responsive to external stimuli downstream of the *At*GRP7 autoregulatory circuit.

## Background

*At*GRP7 (*Arabidopsis thaliana *glycine-rich RNA-binding protein 7) is an RNA-binding protein with an N-terminal RNA recognition motif and a C-terminal glycine-rich domain. It is under control of the circadian clock and has been implicated in stress responses and floral transition [1-6].

The circadian clock is an endogenous timekeeping device that provides the organism with an approximately 24-hour time. The core clockwork comprises transcriptional feedback loops with positively and negatively acting proteins that directly or indirectly regulate their own expression and thus generate their own 24-h rhythm [7-9]. The core oscillator is composed of interconnected morning and evening loops. In the morning loop, the two MYB-type transcription factors CCA1 (CIRCADIAN CLOCK ASSOCIATED 1) and LHY (LATE ELONGATED HYPOCOTYL) activate two pseudo response regulators, *PRR7 *and *PRR9 *that feed back to repress *CCA1 *and *LHY *[10-12]. In the evening loop, TOC1 (TIMING of *CAB *EXPRESSION 1) represses *GI *(*GIGANTEA*) which in turn contributes to *TOC1 *activation [13]. In shoots, these two loops are interlocked through reciprocal regulation of CCA1/LHY and TOC1 [14-18].

The core oscillator imparts rhythmicity on downstream transcripts to generate output rhythms. Among those transcripts is *AtGRP7 *which oscillates with a peak in the evening and is directly controlled by the CCA1 and LHY clock proteins [2,14,19,20]. Notably, *At*GRP7 itself influences the oscillations of its own transcript at the post-transcriptional level [20,21]. *At*GRP7 binds to its own pre-mRNA and promotes the formation of an alternatively spliced transcript that retains part of the intron including a premature termination codon [22,23]. This unproductively spliced transcript form is short-lived and is degraded via the Nonsense-mediated decay (NMD) pathway [24]. Apart from this *At*GRP7 also influences the *AtGRP8 *transcript encoding a related RNA-binding protein. This negative feedback loop is thought to represent a slave oscillator as part of clock output signalling [25,26].

RNA-binding proteins are involved in almost all aspects of RNA metabolism. Upon transcription and throughout their life, mRNAs are bound by a suite of proteins that define pre-mRNA processing, lifetime, export from the nucleus and translation [27,28]. In higher plants, RNA-binding proteins perform a crucial role in key developmental processes such as floral transition and flower development or stress tolerance [29-31]. The targets of these RNA-binding proteins and their mode of action are known in only a few cases, however [32,33].

In order to obtain insights into cellular processes *At*GRP7 may be involved in we set out to globally identify potential *At*GRP7 target transcripts. About 300 transcripts were found to be differentially expressed in plants constitutively overexpressing *At*GRP7 (*At*GRP7-ox). About one third of these are controlled by the circadian clock and they show a certain bias towards specific circadian phases. Furthermore, transcripts associated with responses to stress and to abiotic or biotic stimuli were prevalent. Monitoring for enrichment of gene sets revealed that components associated with various aspects of RNA metabolism predominate among transcripts with higher abundance in *At*GRP7-ox plants.

## Results

### Identification of genes differentially expressed in plants constitutively over-expressing *At*GRP7

Transcripts regulated by the RNA-binding protein *At*GRP7 are expected to have altered expression levels in transgenic plants constitutively overexpressing *At*GRP7, as observed for endogenous *AtGRP7 *whose abundance is depressed by the elevated *At*GRP7 protein level [20]. Therefore, differences in the mRNA complement of wt and *At*GRP7-ox plants were analyzed on the Affymetrix ATH1 microarray. To filter out any line- or accession-specific effects, independent transgenic lines in the C24 background, line RS13 [20], and in the Col background, line G [6], were assayed. Transgenic lines and the wild types were grown in parallel in long days (16 h light, 8 h darkness) and harvested at the circadian maximum of *AtGRP7 *expression (zt12, zeitgeber time 12, 12 hrs after lights on).

The RankProduct algorithm was employed to compare transcript profiles of the *At*GRP7-ox lines to the respective wild types [34]. Transcripts corresponding to 153 probe sets were present at an elevated level in *At*GRP7-ox plants (Additional file 1) and 161 were present at a reduced level (Additional file 2) with a signal log ratio > 0.6 in all three experiments (*p *< 0.05). Among the transcripts most strongly reduced in *At*GRP7-ox plants was *AtGRP8 *previously shown to be under negative control by *At*GRP7 [20,24], validating the strategy.

### Overrepresentation of circadian transcripts among *At*GRP7 targets

**Figure 1:**
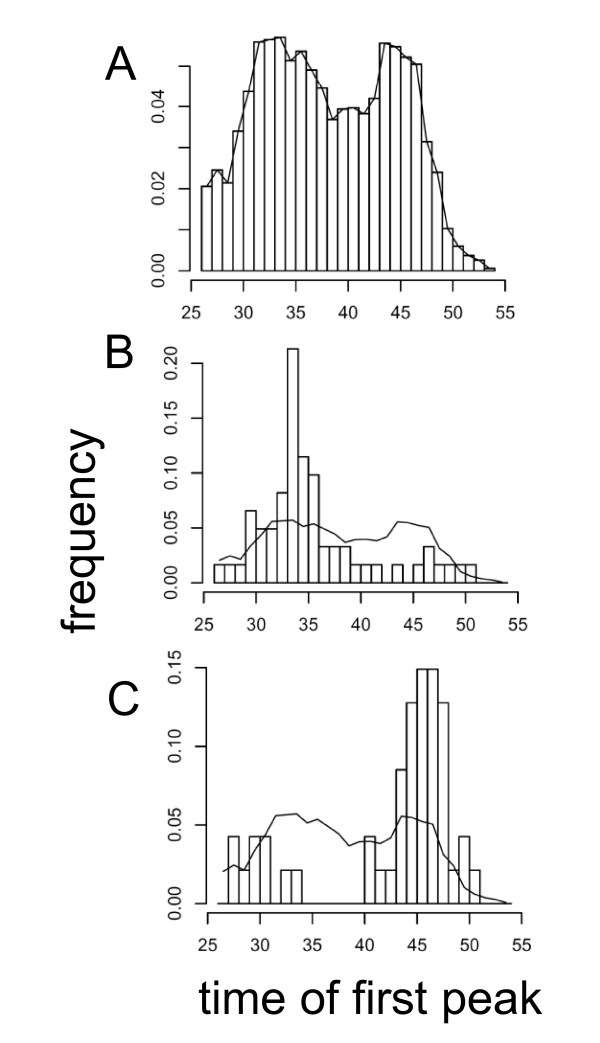
**Phase of transcripts differentially expressed in *At*GRP7-ox**. A) Time of first peak after release from light-dark cycles into continuous light of all transcripts scored rhythmic by COSOPT (pMMC-β < 0.05) according to Edwards et al. (2006). B) Corresponding histogram of genes by Edwards et al. (2006) with reduced levels in *At*GRP7-ox plants. C) Corresponding histogram of genes by Edwards et al. with elevated levels in *At*GRP7-ox plants.

To determine the proportion of rhythmic transcripts among the *At*GRP7 targets, we compared the differentially expressed genes to a published dataset scoring 15.4% of the Arabidopsis genes as circadian-regulated [35]. In this experiment, eight-day-old seedlings were entrained in 12-hr-light/12-hr-dark cycles before transfer to constant light and harvested at 4-hr-intervals, starting 26 h after the last dark-light transition. Thus, subjective dawn corresponds to zt24 and zt48, respectively, and subjective dusk corresponds to zt36. *AtGRP7*, also named *CCR2 *(*COLD AND CIRCADIAN REGULATED 2*), peaked around zt36 in this experiment. Among the transcripts we found to be differentially expressed in *At*GRP7-ox plants rhythmic transcripts are significantly enriched. 47 of the 153 probe sets with elevated levels in *At*GRP7-ox, corresponding to 30.7% (*p *= 5.27E+7) (Additional file 1) and 61 of the 161 probe sets with reduced levels corresponding to 37.9% (*p *= 7.91E+13) (Additional file 2) are among those classified as rhythmic in the Edwards dataset. Edwards and coworkers have shown a nearly uniform distribution of the peaks across all time points (Figure 1A) implicating a complex network downstream of the core oscillator in conveying different phases upon clock-controlled output genes. Notably, the genes differentially expressed in *At*GRP7-ox are biased toward specific circadian phases. A large fraction of transcripts with reduced level in *At*GRP7-ox mainly peaks between zt30 and zt35, in the second half of the subjective day (Figure 1B). This suggests that *At*GRP7 has a mostly negative effect on transcripts oscillating with a similar phase. Conversely, transcripts upregulated in *At*GRP7-ox mainly peak between zt44 and zt50 in the Edwards dataset (Figure 1C), i.e. towards the end of the subjective night and thus in antiphase to *AtGRP7*.

**Figure 2:**
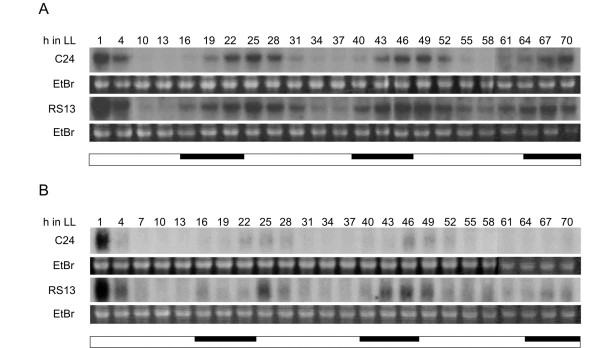
**Influence of *At*GRP7 overexpression on rhythmic target transcripts**. C24 wt and the *At*GRP7-ox plants RS13 were grown in long days for two weeks, transferred to continuous light and harvested at 3-h intervals for three days. The RNA gel blots were hybridized with an *STH *probe (A) and an *HYH *probe (B). A representative northern blot of two independent time courses is shown. The ethidium bromide-stained agarose gel is shown to confirm equal loading. The inserted dark bar indicates subjective night.

To determine how *At*GRP7 affects rhythmic downstream genes, oscillations of selected candidate target transcripts were compared between wt and *At*GRP7-ox plants under free-running conditions. Plants were grown in long days for two weeks and subsequently transferred to continuous light and harvested at 3-h intervals for three days. *SALT TOLERANCE HOMOLOGUE *(*STH*) encoding a B-box zinc finger protein [36] which shows a mean SLR of 1.5 in all three *At*GRP7-ox vs. wt comparisons at zt12 oscillates with a maximum around subjective dawn both in wt and *At*GRP7-ox plants (Figure 2A). In the *At*GRP7-ox plants, the *STH *peak is higher and broader while the phase is maintained. *HY5-HOMOLOG *(*HYH*) encoding a bZip transcription factor involved in phyB signalling [36] which shows a mean SLR of 1.4 in all three *At*GRP7-ox vs. wt comparisons at zt12 (Figure 2B) oscillates at a higher level in *At*GRP7-ox plants.

### Characterization of non-circadian transcripts among *At*GRP7 targets

The observation that one third of *At*GRP7 targets are clock-controlled is in line with a function of the *At*GRP7 feedback loop in clock output but the fact that two thirds are not classified as rhythmic [35] points to additional processes *At*GRP7 may be involved in. The distribution of genes into ontology categories corresponding to plant GOSLIM revealed that the categories "response to stress" and "response to abiotic or biotic stimulus" were significantly enriched among transcripts with reduced levels in *At*GRP7-ox plants (*p *≤ 1.1E-2 and *p *≤ 1.1E-3, respectively) and were also prevalent among transcripts with elevated levels (Table 1, table 2). Further, significant enrichment among transcripts with reduced abundance in *At*GRP7-ox was found for transcripts upregulated by the phytohormones methyl jasmonate (*p *≤ 3.2E-8) and abscisic acid (*p *≤ 1.8E-6).

**Table 1:** GOSLIM categorization in biological processes of genes expressed at reduced levels in *At*GRP7-ox plants.

	n genes selection	expected frequencies	log enrichments	*p *values enriched	*p *values depleted
other physiological processes	22	22.4	-0.025	1	1
response to stress	15	5.2	1.517	1.14E-03	1
response to abiotic or biotic stimulus	16	7.1	1.186	1.05E-02	1
signal transduction	3	6.2	-1.045	1	1
other cellular processes	2	7.4	-1.884	1	0.297
other biological processes	12	13.8	-0.202	1	1
biological process unknown	63	68.8	-0.128	1	1
other metabolic processes	59	53.1	0.153	1	1
developmental processes	4	5.1	-0.349	1	1
cell organization and biogenesis	4	4.0	-0.007	1	1
transcription	10	12.2	-0.282	1	1
protein metabolism	21	23.8	-0.183	1	1
DNA or RNA metabolism	3	4.3	-0.524	1	1
transport	16	12.4	0.369	1	1
electron transport or energy pathways	7	8.3	-0.248	1	1

**Table 2:** GOSLIM categorization in biological processes of genes expressed at elevated levels in *At*GRP7-ox plants.

	n genes selection	expected frequencies	log enrichments	*p *values enriched	*p *values depleted
other physiological processes	21	21.3	-0.018	1	1
response to stress	9	4.9	0.854	0.425	1
response to abiotic or biotic stimulus	12	6.7	0.845	0.252	1
signal transduction	7	5.9	0.251	1	1
other cellular processes	8	7.0	0.189	1	1
other biological processes	13	13.1	-0.013	1	1
biological process unknown	59	65.4	-0.149	1	1
other metabolic processes	56	50.4	0.151	1	1
developmental processes	4	4.8	-0.276	1	1
cell organization and biogenesis	3	3.8	-0.349	1	1
transcription	11	11.6	-0.071	1	1
protein metabolism	23	22.7	0.021	1	1
DNA or RNA metabolism	5	4.1	0.287	1	1
transport	15	11.8	0.349	1	1
electron transport or energy pathways	8	7.9	0.018	1	1

**Figure 3:**
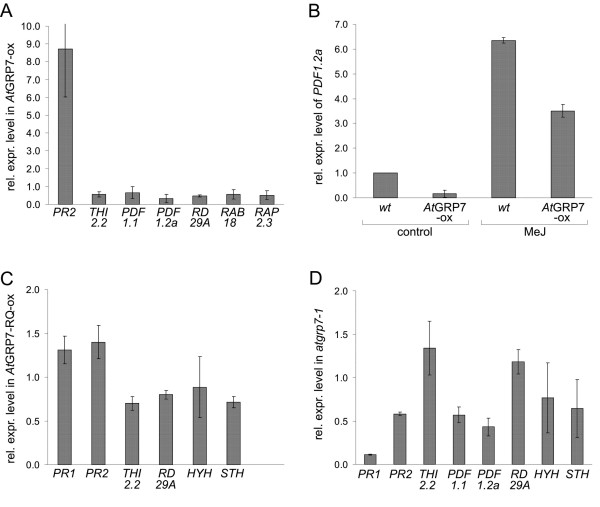
**Analysis of selected *At*GRP7 candidate target transcripts in transgenic lines with elevated or reduced *At*GRP7 levels**. A) *At*GRP7-ox and wt plants were grown in LDs and harvested around the circadian maximum. Expression levels were determined by qRT-PCR and normalized to *PTB *expression. Shown is the expression in *At*GRP7-ox plants relative to wt. B) Transcript levels of the JA-responsive *PDF1.2a *in MeJ-treated plants. Independent *At*GRP7-ox lines and the corresponding wt plants were grown in liquid culture in LDs and harvested 24 h after MeJ treatment. Expression levels were determined by qRT-PCR and normalized to *PTB*. The level in untreated wt plants is set to 1. C) Mutation of a conserved arginine in the *At*GRP7 RNA recognition motif interferes with regulation of target transcripts. qRT-PCR was performed on RNA from *At*GRP7-RQ-ox and wt plants grown in LDs and harvested at zt12 for the analysis of *PR1*, *PR2*, *THI2.2 *and *RD29A*, and at zt3 for the analysis of *HYH *and *STH*, respectively. Data were normalized to *PTB *and the levels in *At*GRP7-RQ-ox plants are expressed relative to wt. D) qRT-PCR was performed on RNA from the T-DNA line *atgrp7-1 *and Col-0 wt plants grown in LDs and harvested at zt12 for the analysis of *PR1*, *PR2*, *THI2.2*, *PDF1.1*, *PDF1.2a *and *RD29A*, and at zt3 for the analysis of *HYH *and *STH*, respectively. Data were normalized to *PTB *and the levels in *atgrp7-1 *are expressed relative to wt.

To validate a differential expression of these candidate targets, a suite of them was monitored in several independent *At*GRP7-ox plants and the corresponding wt plants using RT-PCR and Realtime PCR on zt12 RNA. The transcripts encoding the pathogenesis-related proteins *PR1*, *PR2 *encoding a β-glucanase and *PR5 *encoding an antimicrobial thaumatin-like protein are present at elevated levels in *At*GRP7-ox plants (Figure 3A, Additional file 3). Because two cold- and ABA-regulated transcripts, *RD29A *(*COR78*) and *RAB18*, were confirmed to have reduced levels in *At*GRP7-ox plants (Figure 3A), we tested another cold-induced transcript, *COR15A*, which was also reduced in *At*GRP7-ox plants (Additional file 3). Transcripts encoding the plant defensins *PDF1.1 *and *PDF1.2a*, *RAP2.3/ERF72/AtEBP *encoding a member of the ethylene response factor subfamily B2 of AP2-domain transcription factors implicated in *PDF1.2a *activation [37,38] and the antimicrobial thionin *THI2.2 *are present at reduced levels in *At*GRP7-ox plants (Figure 3A, Additional file 3).

Because methyl jasmonate (MeJ)-induced transcripts were overrepresented among genes with reduced levels in *At*GRP7-ox plants, we investigated a potential role of *At*GRP7 in the response of the *At*GRP7 target gene *PDF1.2a *to MeJ treatment. *PDF1.2a *accumulated 24 hrs after addition of MeJ both in wt and independent *At*GRP7-ox plants (Figure 3B). *PDF1.2a *levels in MeJ-treated *At*GRP7-ox plants still remained lower than in MeJ treated wt plants. Thus, overexpression of *At*GRP7 does not prevent the response to MeJ but MeJ does not overcome the negative regulation by *At*GRP7.

We have shown previously that site-specific mutation of a single arginine to glutamine within the RNA recognition motif impairs both *in vitro *binding of recombinant *At*GRP7 to its pre-mRNA and *in vivo *function [22]. Therefore we investigated the steady-state abundance of selected putative *At*GRP7 targets in transgenic plants constitutively overexpressing the mutant protein (*At*GRP7-RQ-ox). Real time PCR showed that levels of *PR1*, *PR2*, *THI2.2 *and *RD29A *remained similar to wt levels in plants harvested at zt12 (Figure 3C). For *HYH *and *STH *with a morning peak, plants were harvested at zt3. Again, *HYH *and *STH *levels remained similar to wt levels (Figure 3C). The fact that the candidate targets were affected by high levels of the authentic *At*GRP7 protein but not of the mutant protein indicates that the regulation is based on the *At*GRP7 RNA-binding activity. Nevertheless, this does not unambiguously imply direct binding of *At*GRP7 to these transcripts, as overexpression of regulatory RNA-binding proteins, similar to the overexpression of transcription factors leads to direct and indirect effects on the transcriptome. To begin to understand how *At*GRP7 may influence the differentially expressed transcripts we assessed their steady-state abundance in the *atgrp7-1 *T-DNA insertion line that lacks *At*GRP7 [4]. *PR1 *and *PR2 *transcript levels were reduced in *atgrp7-1*, suggesting that their expression closely correlates with the *At*GRP7 level (Figure 3D).

Also *HYH *and *STH *levels were weakly reduced at the time of their circadian maximum. The levels of *THI2.2 *and *RD29A *remained mostly unchanged and levels of *PDF1.1 *and *PDF1.2a *were reduced in *atgrp7-1*, suggesting either that elevated *At*GRP7 levels have a slightly negative effect but reduced *At*GRP7 levels are not sufficient to cause their upregulation or that they are influenced indirectly.

### Analysis of gene set enrichment

Overall, changes in expression of most candidate target genes were moderate (Additional file 1, Additional file 2). Therefore we subjected the expression data to PAGE (parametric analysis of gene set enrichment) [39], an improved tool to analyze overrepresentation of groups of genes that employs predefined gene sets. It relies on the assumption that differential expression manifests itself more clearly at the level of coregulated genes than at the level of individual genes. Thus, PAGE is a complementary approach to uncover genes differentially expressed with a small fold change (below the cut-off level). Its significance comes from the possibility to detect entire gene sets, with narrower definition than GO categories, that are co-ordinately up- or down-regulated to a small degree.

**Figure 4:**
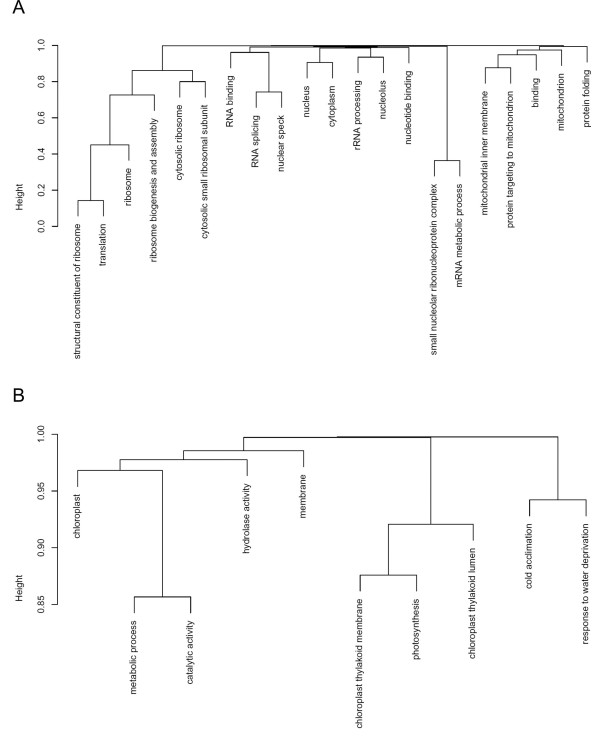
**Gene sets enriched among transcripts differentially expressed in *At*GRP7-ox**. PAGE [39] was used to calculate the Z score for predefined gene sets from the signal log ratio between wt and *At*GRP7-ox plants and to infer statistical significance against standard normal distribution. A) Agglomerative clustering of groups of genes expressed at a higher level in *At*GRP7-ox plants. B) Agglomerative clustering of groups of genes expressed at a reduced level in *At*GRP7-ox plants.

Most prominent among gene sets upregulated in *At*GRP7-ox plants were structural constituents of the ribosome and functions associated with ribosome biogenesis and assembly (Figure 4A, Table 3). The next categories were RNA binding and small nucleolar ribonucleoprotein complexes, followed by rRNA processing, nucleolus and RNA splicing including the SR (serine arginine rich) proteins RSZ22 and RSZ32. This may point to an involvement of the RNA-binding protein *At*GRP7 in the modulation of RNA processing and translational activity. Two transcripts encoding proteins participating in pre-mRNA splicing, the snRNP core protein D1 and the U5 snRNP helicase (Additional file 1), were confirmed to be expressed at higher levels in independent *At*GRP7-ox lines (data not shown). Transcripts with reduced abundance in *At*GRP7-ox comprise functions associated with chloroplasts (Figure 4B, Table 4). This may relate to the observation of a slightly reduced chlorophyll content in *At*GRP7-ox plants (Streitner, unpublished).

**Table 3:** Gene sets enriched among transcripts expressed at elevated levels in *At*GRP7-ox plants.

GO term	Z	*p*	*p *adjusted
structural constituent of ribosome	-16.62418	4.66E-62	2.19E-59
translation	-15.1101	1.39E-51	3.26E-49
ribosome	-15.00005	7.34E-51	1.15E-48
ribosome biogenesis and assembly	-12.17341	4.31E-34	5.06E-32
cytosolic ribosome	-12.06825	1.55E-33	1.46E-31
RNA binding	-9.410485	4.94E-21	3.87E-19
small nucleolar ribonucleoprotein complex	-8.51926	1.61E-17	1.08E-15
cytosolic small ribosomal subunit	-7.678024	1.62E-14	8.44E-13
nucleus	-7.240035	4.49E-13	2.11E-11
mitochondrial inner membrane	-7.047071	1.83E-12	7.81E-11
mRNA metabolic process	-6.383831	1.73E-10	6.76E-09
binding	-5.912648	3.37E-09	1.22E-07
nucleotide binding	-5.874369	4.24E-09	1.42E-07
rRNA processing	-5.858142	4.68E-09	1.42E-07
nucleolus	-5.853125	4.82E-09	1.42E-07
RNA splicing	-5.83756	5.30E-09	1.46E-07
protein targeting to mitochondrion	-5.507412	3.64E-08	9.24E-07
cytoplasm	-5.502979	3.73E-08	9.24E-07
mitochondrion	-5.485297	4.13E-08	9.70E-07
protein folding	-5.425586	5.78E-08	1.29E-06
nuclear speckles	-5.229014	1.70E-07	3.64E-06

**Table 4:** Gene sets enriched among transcripts expressed at reduced levels in *At*GRP7-ox plants.

GO term	Z	*p*	*p *adjusted
chloroplast	-11.5152	1.11E-30	5.20E-28
chloroplast thylakoid membrane	-9.640974	5.37E-22	1.26E-19
cold acclimation	-7.550362	4.34E-14	6.80E-12
chloroplast thylakoid lumen	-6.959285	3.42E-12	3.44E-10
membrane	-6.949736	3.66E-12	3.44E-10
hydrolase activity	-6.410412	1.45E-10	1.14E-08
response to water deprivation	-5.926906	3.09E-09	2.07E-07
metabolic process	-5.873516	4.27E-09	2.51E-07
photosynthesis	-5.681972	1.33E-08	6.26E-07
catalytic activity	-5.316148	1.06E-07	4.53E-06

## Discussion

Transcript profiling has identified about 300 transcripts with altered expression in transgenic lines overexpressing the clock regulated RNA-binding protein *At*GRP7. Gratifyingly, among the transcripts most strongly reduced in *At*GRP7-ox plants is *AtGRP8 *previously shown to be under negative control by *At*GRP7 [20], validating the strategy used to identify candidate targets.

One third of the differentially expressed genes are controlled by the circadian clock, in line with the proposed function of the *At*GRP7 feedback loop as a slave oscillator in clock output [40,41]. Binding of the morning-phased LHY and CCA1 clock proteins to the promoters of some morning-specific genes shows that rhythmic transcripts can be directly controlled by the core oscillator proteins [14]. Other oscillating transcripts presumably are regulated via signalling intermediates that receive timing cues from the circadian clock and in turn convey rhythmicity upon downstream transcripts [42]. For example, circadian oscillations of the MYB factor EARLY PHYTOCHROME RESPONSIVE 1 (EPR1) are controlled by CCA1 and LHY [43]. EPR1 negatively autoregulates, presumably at the transcriptional level and moreover activates the morning-specific *LHC *(*LIGHT HARVESTING CHLOROPYLL BINDING PROTEIN*) genes. Thus, EPR1 may represent a slave oscillator downstream of the core oscillator that contributes to a phase-specific transcriptional program [43]. *At*GRP7 is the first example of a molecular slave oscillator that autoregulates at the posttranscriptional level. Notably, the rhythmic transcripts that are affected by *At*GRP7 overexpression show a distinct phase bias: The up-regulated transcripts preferentially peak around dawn, roughly opposite to the *At*GRP7 peak abundance whereas the down-regulated transcripts preferentially peak at the end of the day (Figure 1). In accordance with this, the morning-phased *STH *and *HYH *transcripts show higher peak levels in an extended time course over three days in LL (Figure 2). Previously, we have found that the *AtGRP8 *transcript, which cycles in phase with *AtGRP7*, is strongly downregulated in *At*GRP7-ox plants but also retains rhythmicity over three days in LL [22]. To begin to understand the relation between the core oscillator and the *At*GRP7 slave oscillator and their respective downstream transcripts, we monitored recently published datasets for the phase distribution of transcripts controlled by TOC1 and LHY with reference to the Edwards dataset [35,44,45]. Transcripts that are elevated in TOC1-ox plants at zt16 in 16 h light-8 h dark cycles [45] peak in the second half of the night, i.e. at time points opposite to *TOC1 *itself (peak at zt36), and transcripts with reduced abundance have a more uniform phase distribution throughout the light phase with a bias towards the evening, similar to *TOC1 *(Additional file 4). From the datasets comparing *lhy *plants expressing elevated *LHY *levels grown in 8 h light-16 h dark cycles to wt we chose zt0 when *LHY *peaks [44]. Of 1503 transcripts elevated in *lhy*, 348 are rhythmic with peaks roughly opposite to *LHY *itself (Additional file 4). Of 1748 transcripts reduced in *lhy*, 255 are rhythmic with a broad distribution during the night and around dawn. Thus, both *At*GRP7 and TOC1 with preferential expression in the evening as well as LHY with a dawn peak have a bias towards negatively affecting the abundance of similarly phased transcripts and positively affecting the abundance of oppositely phased transcripts. To obtain a detailed picture of the RNA networks controlled by core and slave oscillators, respectively, further transcript profiling of plants mis-expressing the components harvested under identical photoperiods around the clock will be required.

*PR1*, *PR2 *and *PR5 *which are expressed at elevated levels in independent transgenic *At*GRP7-ox lines are associated with salicylic acid (SA)-mediated defence pathways [46]. Conversely, *PDF1.2a *encoding a plant defensin, a target of jasmonic acid and ethylene signalling, and *RAP2.3 *encoding an ethylene response factor implicated in *PDF1.2a *activation are expressed at reduced levels in *At*GRP7-ox. Thus, high *At*GRP7 levels correlate with increased expression of SA-responsive *PR *transcripts and decreased expression of the JA-responsive transcripts. Antagonisms between the SA and JA pathways were observed during defence responses [47]. Notably, the *atgrp7-1 *mutant lacking *At*GRP7 shows increased susceptibility to *Pseudomonas syringae *DC3000 [4]. *At*GRP7 is ADP-ribosylated by the *Pseudomonas syringae *type III effector protein HopU1. This modification depends on the conserved Arginine residue that is crucial for RNA binding activity and *in vivo *function and is suggested to interfere with a defence-related function of *At*GRP7 [4,22]. Whether the elevated levels of *PR1*, *PR2 *and *PR5 *in *At*GRP7-ox plants may point to a role of *At*GRP7 in processing of defence-related transcripts remains to be tested. Alternatively, the hormonal balance could be altered in these plants and cause a general stress response. In line with this, overexpression of *At*GRP7 entails reduced levels of the JA-responsive *PDF1.2a *but does not prevent its induction by MeJ (Figure 3B). Our data also indicate that transcripts regulated by the phytohormone ABA are prevalent among *At*GRP7 targets. Previously a considerable overlap of the circadian transcriptome with ABA-related genes has been noted [48,49].

The observation that several target transcripts are affected by elevated levels of the authentic *At*GRP7 but not of the *At*GRP7-RQ mutant protein shows that the effect depends on the RNA-binding activity. Negative regulation in *At*GRP7-ox plants but not *At*GRP7-RQ-ox plants has been observed for the endogenous *AtGRP7 *and *AtGRP8 *transcripts [22]. Both are regulated post-transcriptionally via binding of *At*GRP7 to the pre-mRNAs that entails alternative splicing and degradation through NMD [24]. It seems conceivable that *At*GRP7 may interact with similar binding sites in some of the candidate target transcripts, thus controlling their stability or splicing.

So far, a minimal *AtGRP7 *binding site identified in its 3'UTR [23] was not found to be prevalent in the 5'UTRs or 3'UTRs of either the upregulated or downregulated transcripts (Lewinski and Staiger, unpublished). However, computational identification of RNA substrates based on conserved binding motifs is not straightforward because in addition to the sequence context structural features of the RNA are relevant. Thus, programs for RNA sequence alignment have to be informed by structure [50]. To unequivocally demonstrate direct regulation, *in vivo *binding of the targets by *At*GRP7 will have to be demonstrated by precipitating *At*GRP7-containing mRNP particles from transgenic plants expressing epitope-tagged *At*GRP7 and identification of co-precipitated transcripts.

Transcripts that are controlled directly by *At*GRP7 may be affected in the opposite way by reduced levels of *At*GRP7. Several transcripts such as *PR1 *and *PR2 *that show higher levels in *At*GRP7-ox indeed are present at reduced levels in the *atgrp7-1 *T-DNA insertion line [4]. Several other transcripts that are altered in *At*GRP7-ox plants remain at wt levels in *atgrp7-1 *or are even changed in the same direction. This may indicate that they respond to elevated levels of *At*GRP7 but that *At*GRP7 is not limiting. Alternatively, altered steady-state abundance could also be a secondary consequence of *At*GRP7 overexpression, but nevertheless may be biologically meaningful. For example, it could result from changes in transcription rate as a consequence of modulation of activators or repressors. This could be assessed by measuring the effect of a high *At*GRP7 concentration upon promoter-reporter gene constructs.

Constitutive overexpression of *At*GRP7 promotes the transition to flowering [6]. In the present comparison between *At*GRP7-ox and wt plants transcripts related to flowering time control are not prevalent. Presumably target transcripts associated with the role of *At*GRP7 in floral transition have not been identified in the long-day grown plants because the floral promotive effect of *At*GRP7 manifests itself mostly under short-day conditions [6].

Overall, in the *At*GRP7-ox plants only 1.4% of the transcripts present on the ATH1 Chip are altered, and the changes are moderate (Additional file 1, Additional file 2). In addition to posttranscriptional regulation *At*GRP7 may exert control on downstream targets also at the translational level which is not revealed by transcript profiling. In line with this, gene sets comprising structural components of ribosomes and functions associated with ribosome biogenesis and assembly are enriched among transcripts elevated in *At*GRP7-ox plants. Notably, in *Chlamydomonas reinhardtii *the clock-regulated RNA-binding protein CHLAMY1 has been shown to repress translation of enzymes involved in CO_2_- and N-metabolism [51,52]. That RNA-binding proteins affect multiple facets of post-transcriptional control is not without precedent: Polypyrimidine tract-binding protein, also known as hnRNP I, binds pyrimidine-rich regions in introns to regulate alternative splicing but also is responsible for time-of-day dependent degradation of the mRNA encoding the mammalian clock gene *Period2 *[53].

## Conclusion

**Figure 5:**
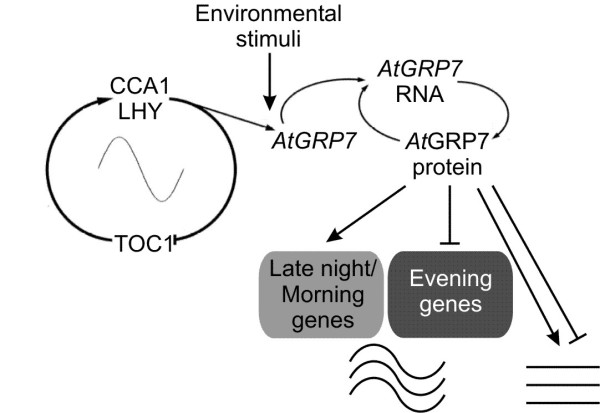
**Conceptual model of the *At*GRP7 feedback loop**. *AtGRP7 *transcript oscillations are under control of the core oscillator [14,60]. Furthermore, *AtGRP7 *expression is influenced by environmental stimuli including cold. *At*GRP7 itself negatively autoregulates at the post-transcriptional level. Downstream targets of *At*GRP7 include circadian transcripts that are affected in a time-of-day dependent manner: *At*GRP7 negatively affects steady-state abundance of evening-phased transcripts and positively affects steady-state abundance of late night/morning genes. Further, transcripts responsive to environmental signals are prevalent among *At*GRP7 targets. Arrows indicate activation, blunt lines indicate negative interaction.

Expression of the RNA-binding protein *At*GRP7 is trigged by the circadian clock. In turn, it affects accumulation of rhythmic transcripts in a time-of-day dependent manner: Transcripts peaking in the evening like *At*GRP7 itself mainly are expressed at reduced levels in *At*GRP7-ox plants whereas transcripts peaking in the morning mainly are expressed at elevated levels (Figure 5). Further, *At*GRP7 directly and indirectly affects a suite of other transcripts including hormone responsive and pathogenesis-related and cold-regulated transcripts. Based on these findings, *At*GRP7 is placed in clock output signalling, transducing timing information from the circadian clock upon downstream targets. Additionally, *At*GRP7 that itself is influence by external stimuli including cold appears to be embedded in an environmental response network (Figure 5).

## Methods

### Plant growth and treatment

The genotypes used were C24, RS13 (*At*GRP7-ox in C24 background) [20], Col, D and G (*At*GRP7-ox in Col background) [22], *At*GRP7RQ-ox [22] and the T-DNA insertion line *atgrp7-1 *[4,6]. Seeds were surface-sterilised, stratified at 4°C for two days, germinated and grown on half-strength MS medium [54] supplemented with 0.5% sucrose and 0.5 g MES/l in long days (16-hr light/8-hr dark cycles) at 20°C. After about ten days seedlings of comparable size were transferred to MS plates without sucrose.

For JA treatment, seeds were germinated in liquid half-strength MS medium supplemented with 0.5% sucrose and 0.5 g MES/l and incubated on a rotary shaker in long days. After 12 days 50 μM MeJ was added and control samples were treated with 0.2% ethanol. Plants were harvested after 24 h.

### RNA isolation for transcript profiling on microarrays

The aerial part of plants with about eight true leaves were harvested at zt12, the time of the circadian maximum of *AtGRP7 *transcript abundance. RNA was isolated using Trizol. Total RNA was treated with DNaseI and further purified using the RNeasy kit (Qiagen, Hilden, Germany).

### Array hybridization

Synthesis of cDNA and biotinylated cRNA were performed as recommended by Affymetrix (Santa Clara, USA). Total RNA (20 μg) was used to prepare cDNA with SuperscriptII Reverse transcriptase (Invitrogen) according to the manufacturer's instructions with an oligo(dT)_24_-T7 oligonucleotide (GGCCAGTGAATTGTAATACGACTCACTATAGGGAGGCGG(dT)_24_). The cDNA was subjected to *in vitro *transcription in the presence of 2 mM of each biotin-11-CTP and biotin-16-UTP (ENZO Life Sciences, Farmingdale, NY) with the MegaScript High Yield Transcription Kit (Ambion, Austin, TX). After purification of the cRNA on RNeasy columns (Qiagen, Hilden, Germany), 15 μg of cRNA was fragmented in a volume of 40 μl, denatured for 5 min at 99°C and hybridized to the arrays for 16 h. Washing and detection of labelled cRNA with streptavidin-phycoerythrin were performed according to the manufacturer's instructions. The arrays were scanned using Affymetrix 3000 7G confocal scanner. Affymetrix Arabidopsis ATH1 GeneChips(r) were used throughout the experiment (Affymetrix, Santa Clara, CA). The exact list of probes present on the arrays can be obtained from the manufacturer's website http://www.affymetrix.com. Analysis was based upon annotations compiled by TAIR (http://www.arabidopsis.org, version 2007-5-2).

### Data analysis

Raw data were processed with MAS (Microarray suite) 5.0 from Affymetrix. Signal values were derived from Affymetrix *.cel files using GCRMA [55]. All data processing was performed using the statistic language R (version 2.6.2) that is freely available at http://www.r-project.org/[56].

Coefficients of variation (*cv*) between replicates as a quantitative measure of data quality and consistency between replicates were calculated as described previously [57]. Differentially expressed genes were identified using the *RankProd *package in R [34] that inherently corrects for multiple testing. Probe sets were called significantly differentially expressed when *q *< 0.05. To enrich for biologically relevant changes, only probe sets with a minimal fold change of 1.5 were selected. Differentially expressed genes were grouped into collapsed functional gene ontology categories (GOSLIM, obtained from http://www.arabidopsis.org). Furthermore, enrichment of detailed GO categories (obtained from TAIR) was tested. In this case, multiple-testing correction was according to [58] with a critical *p*-value of 1E-2. Grouping according to preferred phase of circadian expression was based on data from Edwards et al. [35]. Grouping according to phytohormone regulation was based on data from Nemhauser et al. [59]. The significance of enrichment was estimated based on the hypergeometric test and multiple-testing correction according to Bonferroni. PAGE (parametric analysis of gene set enrichment) was performed as described [39] using a critical *p*-value of 1E-6 after multiple testing correction according to Benjamini and Hochberg [58].

### RNA analysis

Isolation of total RNA and hybridization of RNA gel blots were performed as described [60]. For semiquantitative RT PCR, retrotranscribed RNA was amplified with Taq Polymerase. To determine the linear range of amplification for each primer pair, samples were withdrawn after 24, 26, 28, 30, 32 and 34 cycles. PCR products were separated on agarose gels and either visualized by Ethidium-bromide staining or transferred to a nylon membrane and hybridized with radiolabeled cDNA probes.

For Real time PCR, duplicate samples were analysed in a MJ research Opticon DNA Engine. Total RNA was treated with DNaseI and reverse-transcribed using Superscript II (Invitrogen). 20 ng of retrotranscribed RNA was amplified with the Eppendorf Real MasterMix kit using an initial denaturation step of 2 min, followed by 45 cycles of 20 sec at 94°C, 30 sec at 60°C and 40 sec at 68°C. C_T _values were determined and relative expression levels for the analyzed transcripts were calculated based on non-equal efficiencies for each primer pair [61,62]. Data were normalized to transcripts encoding the translation initiation factor eIF-4A-1 (At3g13920), PTB (At3g01150) and PPR (At5g55840) [63]. Shown are the mean expression levels +/- s.d.. The absence of amplification products from genomic DNA was confirmed in non-retrotranscribed controls. Primers are listed in Additional file 5.

## Authors' contributions

C.S. and C.K. performed experiments, L. H. analyzed the Affymetrix data set and performed data mining, D.S. designed the experiments and wrote the paper. All authors read and approved the final manuscript.

## Supplementary Material

Additional file 1 **Transcripts present at an elevated level in *At*GRP7-ox plants**. Additional file 1 contains a list of the transcripts present at an elevated level in *At*GRP7-ox plants with a signal log ratio > 0.6. Transcripts classified as rhythmic according to Edwards et al. (2006) are marked by an asterisk. The median cv (coefficient of variation) values between the C24 replicates and the RS13 replicates were 2.4% and 2.7%, respectively, demonstrating the high quality of the data. Click here for file

Additional file 2 **Transcripts present at a reduced level in *At*GRP7-ox plants**. Additional file 2 contains a list of the transcripts present at a reduced level in *At*GRP7-ox plants with a signal log ratio < -0.6. Transcripts classified as rhythmic according to Edwards et al. (2006) are marked by an asterisk. Click here for file

Additional file 3 **RT-PCR analysis of selected *At*GRP7 candidate target transcripts in *At*GRP7-ox plants**. Additional file 3 shows a figure with expression data of targets in *At*GRP7-ox and wt plants grown in LDs and harvested around the circadian maximum. RT-PCR products were separated on agarose gels and visualized by Ethidium bromide-staining. The *THI2.2 *transcript was detected by hybridization with a ^32^P labelled probe. The gels show results representative for several independent transgenic lines in the Col or C24 background. Click here for file

Additional file 4 **Phase distribution of transcripts controlled by LHY and TOC1**. Additional file 4 shows diagrams of the phases of LHY and TOC1 target transcripts. A) Phase of transcripts differentially expressed in transgenic plants overexpressing the oscillator component TOC1 (data from [45]). 245 transcripts present at an elevated level in TOC1-ox plants and 160 transcripts present at a reduced level in TOC1-ox plants harvested at zt16 in LDs were interrogated for their first peak after release from light-dark cycles into continuous light in the Edwards dataset of transcript scored rhythmic by COSOPT [35]. B) Phase of transcripts differentially expressed in *lhy *mutants expressing elevated levels of the oscillator component LHY (data from [44]). GC-RMA normalized data for *lhy *and Col plants harvested at zt0 in SDs were downloaded. Transcripts expressed with a signal-log ratio < 1 were interrogated for their first peak after release from light-dark cycles into continuous light in the Edwards dataset of transcripts scored rhythmic by COSOPT [35]. Click here for file

Additional file 5 **PCR primers**. Additional file 5 contains a list of the PCR primers used for transcript analysis by RT-PCR, eal-Time RT-PCR and amplification of hybridization probes. Click here for file
