# Supplementary figures and images for: Global transcript profiling of transgenic plants constitutively overexpressing the RNA-binding protein AtGRP7
Source: BMC Plant Biol. 2010 Oct 14;10:221. doi: 10.1186/1471-2229-10-221 (PMC3017831; doi:10.1186/1471-2229-10-221)

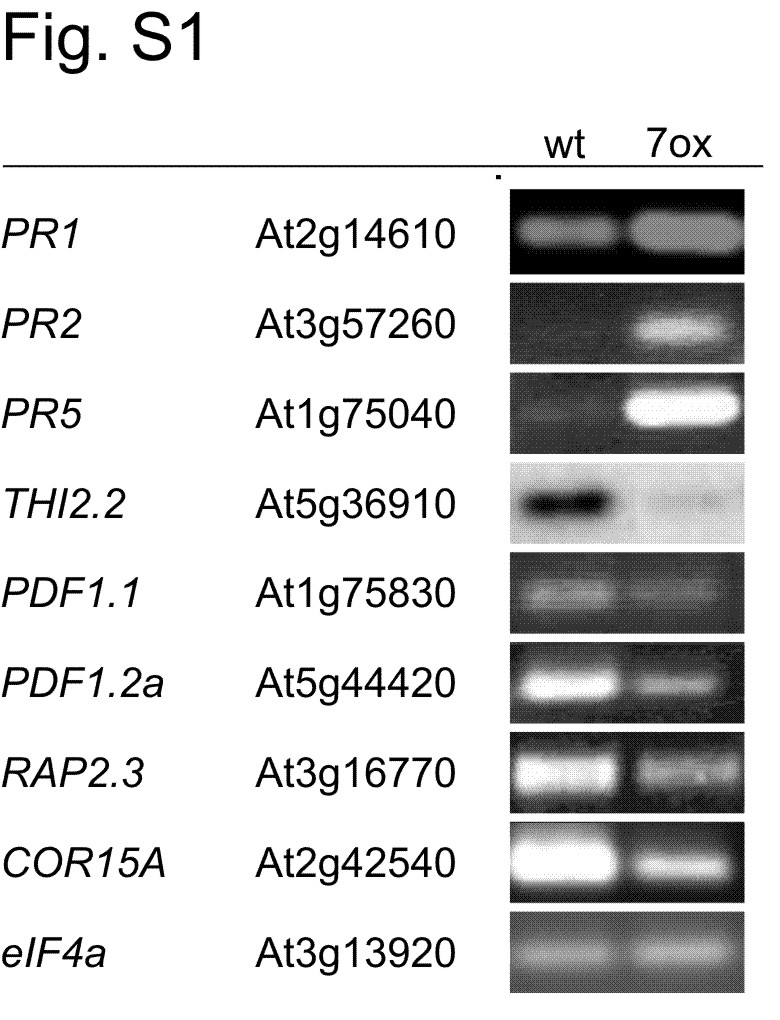

Supplement: Additional file 3 — RT-PCR analysis of selected AtGRP7 candidate target transcripts in AtGRP7-ox plants. Additional file 3 shows a figure with expression data of targets in AtGRP7-ox and wt plants grown in LDs and harvested around the circadian maximum. RT-PCR products were separated on agarose gels and visualized by Ethidium bromide-staining. The THI2.2 transcript was detected by hybridization with a 32P labelled probe. The gels show results representative for several independent transgenic lines in the Col or C24 background. [file 1471-2229-10-221-S3.jpeg]

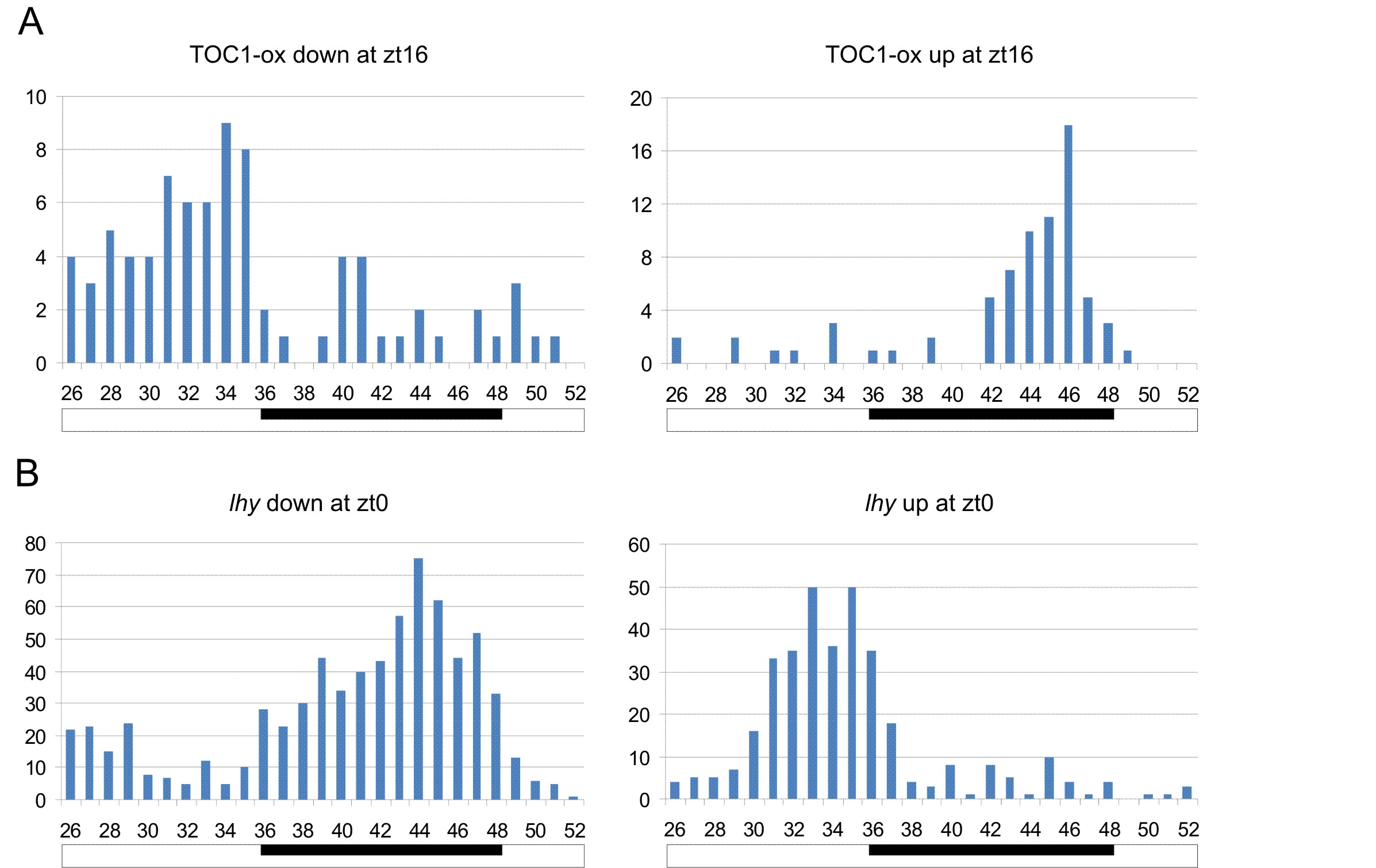

Supplement: Additional file 4 — Phase distribution of transcripts controlled by LHY and TOC1. Additional file 4 shows diagrams of the phases of LHY and TOC1 target transcripts. A) Phase of transcripts differentially expressed in transgenic plants overexpressing the oscillator component TOC1 (data from [45]). 245 transcripts present at an elevated level in TOC1-ox plants and 160 transcripts present at a reduced level in TOC1-ox plants harvested at zt16 in LDs were interrogated for their first peak after release from light-dark cycles into continuous light in the Edwards dataset of transcript scored rhythmic by COSOPT [35]. B) Phase of transcripts differentially expressed in lhy mutants expressing elevated levels of the oscillator component LHY (data from [44]). GC-RMA normalized data for lhy and Col plants harvested at zt0 in SDs were downloaded. Transcripts expressed with a signal-log ratio < 1 were interrogated for their first peak after release from light-dark cycles into continuous light in the Edwards dataset of transcripts scored rhythmic by COSOPT [35]. [file 1471-2229-10-221-S4.jpeg]
