# Supplementary material for: Global transcript profiling of transgenic plants constitutively overexpressing the RNA-binding protein AtGRP7
Source: BMC Plant Biol. 2010 Oct 14;10:221. doi: 10.1186/1471-2229-10-221 (PMC3017831; doi:10.1186/1471-2229-10-221)
Supplement: Additional file 5 — PCR primers. Additional file 5 contains a list of the PCR primers used for transcript analysis by RT-PCR, eal-Time RT-PCR and amplification of hybridization probes. [file 1471-2229-10-221-S5.doc]

Primers used for transcript analysis by Real-Time RT-PCR or RT-PCR and generation of hybridization probes.

RAP2.3/ERF72 (At3g16770.1)

for atgtgtggcggtgctattatttcc

rev atacgacgcaatgacatcatcaag

COR15A (At2g42540)

for atggcgatgtctttctcagg

rev ctactttgtggcatccttagc

RD29A (At5g52310)

for tcccaccaaagaagaaactg

rev gaattctttgcttctcgtcg

RAB18 (At5g66400)

for cagctctagctcggaggatg

rev gtagccaccagcatcatatcc

PR5 (At1g75040)

fwd gcaaatatctccagtattcacattc

rev ttaagggcagaaagtgatttcg

PR2 (At3g57260)

for tcatcctcgacgttcccagt

rev tcaagccctgctccagaaac

PR1 (At2g14610)

for atgaattttactggctattctcga

rev ttagtatggcttctcgttcacat

PDF1.1 (At1g75830)

for ctcttgaagcaccgatgg

rev gtgagctgggaagacatagt

PDF 1.2a (At5g44420)

for acgcaccggcaatggtggaagcac

rev ttaacatgggacgtaacagatacac

THI2.2 (At5g36910)

for ccatggctcttgttcaatccagaac

rev gatgtgccacataacttggatc

STH (At2g31380)

for ctcataaagcccttcttatc

rev cacacacatcacattgtatc

HYH (At3g17609)

for aaagtgatgaggagttgttg

rev ctcttgaggcttctatattc

PPR (At5g55840)

for aagacagtgaaggtgcaaccttact

rev agtttttgagttgtatttgtcagagaaag

eIF-4A (At3g13920)

for caacaagtctccctggttatc

rev agcatcctctcatcatcacgg

PTB (At3g01150)

for gatctgaatgttaaggcttttagcg

rev ggcttagatcaggaagtgtatagtctctg
